# Mapping the 12-item World Health Organization disability assessment schedule 2.0 (WHODAS 2.0) onto the assessment of quality of life (AQoL)-4D utilities

**DOI:** 10.1007/s11136-023-03532-9

**Published:** 2023-10-31

**Authors:** Bernice Hua Ma, Gang Chen, Samia Badji, Dennis Petrie

**Affiliations:** 1 Monash Business School Centre for Health Economics, Caulfield East, Australia; 2 Centre of Research Excellence in Disability and Health, Parkville, Australia

**Keywords:** Disability, Quality of life, AQoL-4D, WHODAS, Mapping, Utility

## Abstract

**Purpose:**

The World Health Organization Disability Assessment Schedule 2.0 (WHODAS 2.0) is a widely used disability-specific outcome measure. This study develops mapping algorithms to estimate Assessment of Quality of Life (AQoL)-4D utilities based on the WHODAS 2.0 responses to facilitate economic evaluation.

**Methods:**

The study sample comprises people with disability or long-term conditions (*n* = 3376) from the 2007 Australian National Survey of Mental Health and Wellbeing. Traditional regression techniques (i.e., Ordinary Least Square regression, Robust MM regression, Generalised Linear Model and Betamix Regression) and machine learning techniques (i.e., Lasso regression, Boosted regression, Supported vector regression) were used. Five-fold internal cross-validation was performed. Model performance was assessed using a series of goodness-of-fit measures.

**Results:**

The robust MM estimator produced the preferred mapping algorithm for the overall sample with the smallest mean absolute error in cross-validation (MAE = 0.1325). Different methods performed differently for different disability subgroups, with the subgroup with profound or severe restrictions having the highest MAE across all methods and models.

**Conclusion:**

The developed mapping algorithm enables cost-utility analyses of interventions for people with disability where the WHODAS 2.0 has been collected. Mapping algorithms developed from different methods should be considered in sensitivity analyses in economic evaluations.

**Supplementary Information:**

The online version contains supplementary material available at 10.1007/s11136-023-03532-9.

## Introduction

Preference-based quality-of-life data is increasingly collected for economic evaluation studies, such as cost-utility analysis (CUA), to compare value for money and prioritise limited resources. Quality-adjusted life years (QALYs) are the predominant outcome measure in CUAs, calculated using a preference-based multi-attribute utility instrument (MAUI) like EQ-5D [1, 2] and AQoL-4D to measure quality of life [3].

The QALY is the preferred outcome measure used by many government funding bodies, such as the National Institute for Health and Care Excellence (NICE) in the UK [4, 5] and the Pharmaceutical Benefits Advisory Committee (PBAC) in Australia [6]. However, in many disability studies, researchers still prefer to use a non-preference-based disability-specific instrument [7]. A recent review of disability outcome measures identified 20 generic instruments and reported that the World Health Organisation Disability Assessment Schedule (WHODAS) is widely used [8]; The 12-item version of the WHODAS 2.0 has been validated with people with different types of disabilities [9–14]. However, the summary score of WHODAS 2.0 is non-preference-based, which does not permit the construction of QALYs [8]. Mapping analysis estimates a statistical relationship between preference-based and non-preference-based instruments, the "next-best" approach to deriving health state utilities from a non-preference-based instrument [15].

No current study has developed mapping algorithms to generate health utility from WHODAS 2.0. Lokkerbol et al. [16] have estimated an algorithm for mapping WHODAS 2.0 (both 36 and 12 versions) to disability weights (i.e., to calculate Disability Adjusted Life Years) using data from the World Health Organisation (WHO) Multi-Country Survey Study on Health and Responsiveness (MCSS) [17]. But it included only eight out of the 12 questions of WHODAS 2.0 short form. This may impact the accuracy of the prediction if the missing items are significant predictors.

Traditional econometric methods such as ordinary least squares (OLS) and the generalised linear model (GLM) have been commonly used to create mapping algorithms [18, 19]. Specifying the optimal functional form is often difficult when complex non-linear relationships exist between the source and target instruments. Furthermore, the distribution of a MAUI is often skewed, bounded at one, and maybe multinomial, which adds further complexity to identifying the optimal mapping algorithm [20]. Recently, supervised machine learning techniques have increasingly been used in mapping studies as they have the potential to select important predictors and account for non-linear relationships more efficiently and effectively than traditional approaches. Gao et al. [21] used a deep neural network method to develop mapping algorithms from the MacNew Heart Disease Quality of Life Questionnaire onto different country-specific value sets of EQ-5D-5L (*n* = 943). They found that the machine learning technique performed similarly to the traditional econometric methods in three out of the four countries of their sample. Another study by Aghdaee et al. [22] also found that machine learning (e.g., Lasso regression) performed marginally better if not combined with other traditional econometric methods (*n* = 2015). Despite these previous findings, machine learning still has the advantage of determining the nature of the relationships without researchers trying to guess the possible combinations between them or imposing their bias on the results by selecting their preferred functional form. With a larger dataset than the previous studies, the performance of machine learning techniques in our study may improve.

The objective of the study is to derive optimal mapping algorithms from the 12-item version of WHODAS 2.0 (hereafter 'WHODAS') to the Assessment of Quality of Life-4 Dimension (AQoL-4D), which is a validated generic health-related preference-based instrument that is widely used in disability studies [23–26]. This present study also contributes to the mapping literature by comparing results from traditional econometric models to machine learning approaches.

## Methods

This study follows the Mapping onto Preference-based measures reporting standards (MAPS) from the International Society for Pharmacoeconomics and Outcomes Research (ISPOR) to conduct and report mapping analyses [15]. To apply the machine learning techniques, we followed the steps and best practice recommendations from Doupe et al. [27]. All statistical analyses were conducted using Stata 16 except the exploratory factor analysis, which was performed using the EViews software version 12.

### Data and sample

The data were obtained from the 2007 Australian National Survey of Mental Health and Wellbeing (NSMHWB). The 2007 NSMHWB was conducted throughout Australia from August to December 2007 on a national representative sample. Residences of private dwellings were randomly selected using a stratified, multistage area approach, and then one person meeting the age criteria of 16–85 years was selected from the dwelling. There were 14,805 eligible dwellings out of the initial 17,352 selected dwellings due to all residents being out of scope or empty dwellings. Finally, 8841 complete responses were recorded from the face-to-face interviews (60% response rate).

Both the WHODAS and AQoL-4D were included in this survey. Considering WHODAS is commonly used among people with disability, in our study, we constrained the study sample to be people who reported having a disability, regardless of whether they have a current restriction in activities or not (*n* = 3376). We also excluded a small proportion (*n* = 30) if they did not answer all of the WHODAS and the AQoL-4D items. The final study sample consists of 3376 respondents. The detailed sample selection process can be found in the flowchart in the Electronic supplementary materials (ESM) (ESM 1 Fig. S1).

### Instruments

#### Source measure: WHODAS 2.0–12

The WHODAS is a disability-specific instrument in which all items use a five-point ordinal response scale (1 = None, 2 = Mild, 3 = Moderate, 4 = Severe, and 5 = Extreme/Cannot do). This instrument has a recall period of 30 days [28].

The WHODAS captures functioning in six domains, including cognitive (Items 3 and 6), mobility (Items 1 and 7), self-care (Items 8 and 9), getting along (Items 10 and 11), life activities (Item 2) and participation (Items 4 and 5). This study uses the widely used simple scoring method to calculate the WHODAS summary score, which is calculated as a sum of all items' raw scores [9, 14, 24]. The summary scores of the WHODAS, therefore, range from 12 (no disability) to 60 (full disability).

#### Target measure: AQoL-4D

AQoL-4D is a valid generic preference-based quality-of-life instrument [3, 29]. It contains 12 items (each with four response levels) and is evenly grouped into four dimensions (independent living, relationships, mental health and senses). This instrument has a recall period of seven days. The preference weight of AQoL-4D was developed using the time-trade-off approach among the Australian general public. The utilities range from -0.04 (worse than death) to 1 (full health).

More detailed comparisons of the domains and characteristics between the source (i.e., WHODAS) and the target measures (i.e., AQoL-4D) are presented in ESM 1 Table S1.

### Statistical analysis

As previous literature suggested [30, 31], exploratory data analysis was first conducted to understand the degree of conceptual overlap between the two instruments, including Pearson correlation and exploratory factor analysis (EFA). The maximum likelihood factor analysis with a minimum average partial method was used to consider how many factors to retain in the factor analysis. Factors were rotated using the orthoblique promax method [32]. The EFA would provide a clear view of the underlying structures between the two instruments, the information of which is useful for checking the feasibility of conducting a mapping analysis as well as the potential WHODAS item selection when developing mapping algorithms using traditional econometric techniques.

We used a direct mapping approach to predict AQoL-4D from WHODAS items. Indirect response mapping, which has also been used in the mapping literature, particularly when predicting EQ-5D, was not considered. Unlike EQ-5D, in which only responses to five dimensions need to be estimated, to accurately predict AQoL-4D, researchers need to predict responses to 12 dimensions. There is a higher chance of predicting errors; in particular, the AQoL-4D include items not directly captured by WHODAS (see EMS Table).

We used four traditional econometric and three machine-learning techniques to develop mapping algorithms. The four traditional regression techniques have been widely used in the mapping literature [17, 19, 33–36]. They consist of (i) the ordinary least squares (OLS) estimator, which is the most widely used technique in the mapping literature; (ii) the robust MM estimator, which reduces the influence from potential outliers; (iii) the generalised linear model (GLM), which permits different combinations of distribution families and link functions, including non-normal distributions; and (iv) the beta mixture regression model which is suitable for analysing data with a continuous response variable that ranges from 0 to 1 and follows a beta distribution. Recently, the adjusted limited dependent variable mixture models (ALDVMM) have been developed that perform well with EQ-5D data [10, 37]. However, since AQoL-4D does not have a large gap between 1 and the next feasible value as EQ-5D-3L UK tariff does, this method is not applied in our study.

The following two basic model specifications were considered for predicting the AQoL utility ($\mathrm{uAQoL}4\mathrm{D})$ using the item-level responses of WHODAS. Instead of treating a WHODAS item as a continuous variable, it was transformed into five indicator variables corresponding to each of the response levels. Using level 1 as the reference category, the mapping function therefore included four variables for each of the 12 WHODAS items, thereby allowing for the potential non-linear effects across different levels.

1 $$\mathrm{uAQoL}4{\mathrm{D}}_{i}=\alpha +{\sum \beta }_{j}*\mathrm{WHODAS}\_\mathrm{Ite}{\mathrm{m}\_\mathrm{level}}_{ij}$$

2 $$\mathrm{uAQoL}4{\mathrm{D}}_{i}=\alpha +{\sum \beta }_{\mathrm{j}}*\mathrm{WHODAS}\_\mathrm{Ite}{\mathrm{m}\_\mathrm{level}}_{ij}+{\gamma }_{1}*{\mathrm{Sex}}_{i}+{\gamma }_{2}*{\mathrm{Age}}_{i}$$

where $\mathrm{uAQoL}4\mathrm{D}$ represents the predicted utility for the individual $i$. $\mathrm{WHODAS}\_\mathrm{Ite}{\mathrm{m}\_\mathrm{level}}_{ij}$ is the set of binary variables constructed from the response levels of the WHODAS items. For each item, there are five levels. Therefore, four dummies will be included, with the first level serving as a reference level. Age is in years, and Sex is a binary variable equal to 1 for males and 0 for females.

To ensure that the coefficients of the WHODAS items follow a monotonic pattern (i.e., more severe disability levels have larger or equal decrements compared to less severe levels), we imposed additional constraints in traditional regression models. This involved combining item levels or excluding items with positive coefficients during estimation.

In addition, we employed three machine learning techniques: (i) Lasso regression; (ii) Support vector regression (SVR); (iii) Boosted regression (Boosting), all of which can be applied to continuous outcomes. Lasso regression is a method that selects and fits covariates in a model that minimises prediction errors, using the "shrinkage" method that constrains less important parameters towards zero. The key benefit of lasso regression is that it can automatically perform variable selection, providing a simpler model with only the most relevant features. This can help reduce model complexity and improve generalisation performance on new data [37]. In support vector regression (SVR), the main objective is to find a function that best fits the data and minimises errors between the predicted and observed values. It is done by identifying an *n*-dimensional space (hyperplane) that lies at an optimal distance from the data points (aka., support vectors) [38]. Boosted regression enhances accuracy by combining predictions from multiple weaker models via a weighted average. It starts with base learners, which are typically shallow decision trees, and then trains multiple weak learners in an iterative manner. After each weak learner is trained, it combines the trained weak learners using a weighted scheme. Boosted regression excels in dealing with intricate, non-linear dependencies between features and the target variable. The amalgamation of multiple weak learners empowers the final boosted model, imparting robustness and achieving high predictive accuracy [1, 2].

Apart from Lasso regression, where it assumes a linear functional form as we specified the overarching model structure (3), the other techniques are data-driven and do not rely on strong assumptions about the possible function form. The relationships of the variables are determined by the machine learning algorithms. They could be linear or non-linear depending on the patterns and structures present in the dataset.

3 $$\mathrm{uAQoL}4{\mathrm{D}}_{i}=\alpha +{\sum \beta }_{\mathrm{j}}.\mathrm{WHODAS}\_\mathrm{Ite}{\mathrm{m}\_\mathrm{level}}_{ij}+{\sum \lambda }_{\mathrm{j}}.\mathrm{WHODAS}\_\mathrm{Ite}{\mathrm{m}\_\mathrm{level}}_{ij}\#\mathrm{WHODAS}\_\mathrm{Ite}{\mathrm{m}\_\mathrm{level}}_{iq}+{\gamma }_{1}*{\mathrm{Sex}}_{i}+{\gamma }_{2}*{\mathrm{Age}}_{i}$$

where $\mathrm{uAQoL}4\mathrm{D}$ represents the predicted utility for the individual $i$. $\mathrm{WHODAS}\_\mathrm{Ite}{\mathrm{m}\_\mathrm{level}}_{ij}$ is the set of binary variables constructed from the response levels of the WHODAS items. For each item, there are five levels. Therefore, four dummies will be included, with the first level serving as a reference level. Age is in years, and Sex is a binary variable equal to 1 for males and 0 for females.

### Assessing goodness-of-fit

We employed five-fold cross-validation to evaluate goodness-of-fit. The full sample was randomly split into five groups with equal observations, where 80% of the data were used for algorithm development and the remaining 20% for performance assessment. This process was repeated five times, with each group used four times for estimation and once for validation. The optimal model and method were selected based on the best goodness-of-fit test result from the pooled estimated errors in the absence of external validation data.

Three goodness-of-fit statistics were used: (i) the mean absolute error (MAE), (ii) the root mean square error (RMSE), and (iii) the intraclass correlation (ICC) using a random effect model. We selected MAE as the primary criterion because the MAE is the most natural and unambiguous measure of the average error magnitude, while the RMSE places more weight on outliers [39]. Additionally, we calculated the percentages of observations for which the difference between the observed and predicted values was larger than 0.03, which was performed in previous studies [36, 40]. We also considered the performance of predicting the lower and upper bound of AQoL-4D when selecting the optimal algorithm.

To use the predicted utility scores in real life, it is important to ensure that the final predictions fall within the theoretic boundary of the targeting instrument [19, 34, 41]. In our studies, the lower and upper values are truncated at − 0.04 and 1, respectively, to ensure the predicted value falls into the theoretical range.

The final mapping algorithms were developed using full-sample observations and are based on the method and model that perform the best in the five-fold cross-validation.

### Assessing mapping performance among different disability restriction levels

It is a common observation from previous mapping studies that mapping functions could perform relatively poorly in predicting lower utilities [19, 36, 42]. We therefore reported the performance of different mapping algorithms on people with different disability restriction levels. Results from this sub-sample analysis provide useful information for users to better understand the direction and magnitudes of potential prediction bias.

We divided our sample into five subgroups, including people with profound or severe core restrictions, moderate core restrictions, mild core restrictions, school/employment restrictions, or no specific restrictions. We then performed two types of subgroup analysis. First, we calculated the goodness-of-fit statistics for each subgroup. In addition, we calculated the between-group margins of error in the predicted differences of average utilities between a particular subgroup with restrictions versus the subgroup with no specific restrictions.^1^ This was conducted to assess how accurately the mapping algorithms capture the difference between different severity levels, which could represent incremental utilities across different disease states in economic evaluations.

## Results

### Descriptive analysis

Table 1 presents the descriptive characteristics of our study sample. Among 3376 people with disability in our sample, the mean age was 54 (SD = 19), and 51% were female. About 3% reported profound core activity restrictions, 58% reported having no specific restrictions, and 7% and 19% reported having excellent physical or mental health, respectively. The mean AQoL-4D utility was 0.70 (SD = 0.26), and the mean WHODAS 2.0–12 total score was 18 (SD = 7).

**Table 1:** Participant characteristics (*n* = 3376)

	Total
Age (Mean, SD)	54.0 (18.63)
Sex (*N*, %)	
Female	1733 (51.33)
Male	1643 (48.67)
Disability status (*N*, %)	
Profound core activity restriction	101 (2.99)
Severe core activity restriction	193 (5.72)
Moderate core activity restriction	470 (13.92)
Mild core activity restriction	184 (5.45)
School/employment restriction	473 (14.01)
No specific restriction	1955 (57.91)
Self-assessed physical health (*N*, %)	
Excellent	231 (6.84)
Very good	901 (26.69)
Good	1246 (36.91)
Fair	728 (21.56)
Poor	270 (8.00)
Self-assessed mental health (*N*, %)	
Excellent	641 (18.99)
Very good	1,094 (32.41)
Good	1,041 (30.84)
Fair	494 (14.63)
Poor	105 (3.11)
Not known	< 10
AQoL-4D utility score, mean (SD)	0.70 (0.26)
WHODAS summary score, mean (SD)	17.97 (6.97)

*AQoL-4D* Assessment of Quality of Life-4 dimensions, *WHODAS 2.0–12* World health organisation disability assessment scheme version 2, 12 items version

**Fig. 1:**
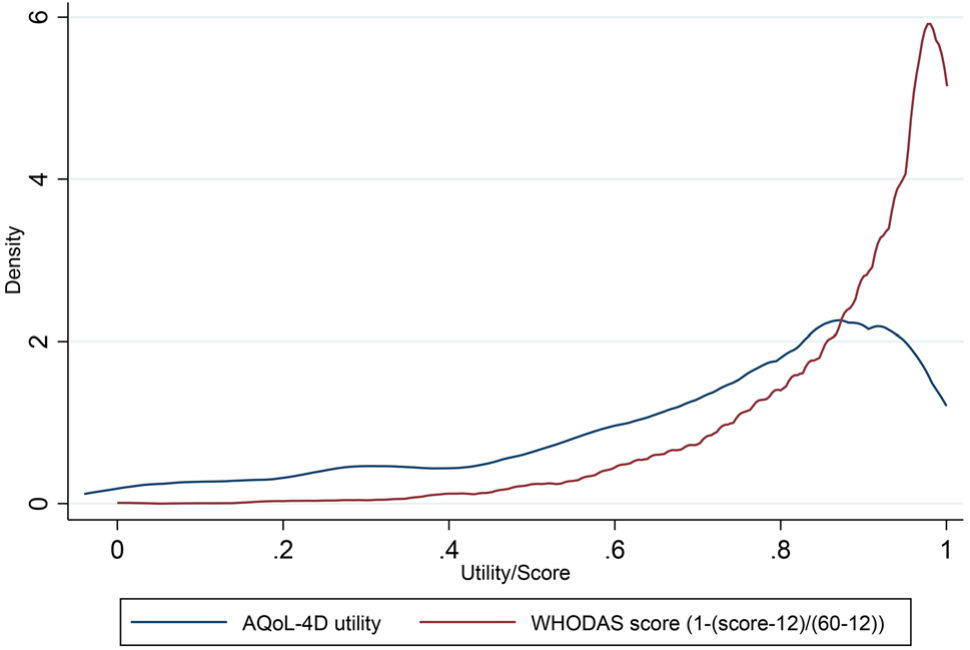
The distribution of the AQoL-4D utilities and the re-scaled WHODAS summary scores

The distribution of the AQoL-4D utilities and the WHODAS summary scores is shown in Fig. 1. They are both negatively skewed and non-normal. The comparison of the two instruments can be found in ESM 1 Table S1. We found that the AQoL-4D utilities and the WHODAS total score had a moderate to strong correlation (*r* = − 0.68), all significant at the 5% level (ESM 1 Table S2), and two factors were identified in the EFA—physical health and psychosocial health (ESM 1 Table S3), which demonstrated good overlaps between the two instruments.

### Mapping results

Table 2 compares the goodness-of-fit statistics for the best five models. The first three columns show the predicted mean, the minimum and maximum values of AQoL-4D, while the last four columns report MAE, RMSE, ICC and the percentage of margin of error in predicted utility smaller than 0.03. All methods generally predicted the mean utility well, but MM and SVM overestimated it. The MM method in Model 2 (age and sex adjusted) had the lowest MAE value (MAE = 0.1325) and the second-highest percentage of margin of error smaller than 0.03 (18.78%). The MM method in Model 1, without adjusting to age and sex, had a similar MAE (0.1326) and the highest percentage of margin of error smaller than 0.03 (18.84%). The full results for all models and methods are presented in ESM Table S4.

**Table 2:** Goodness-of-fit results from five-fold validation samples (*n* = 3376)

Model specification	Descriptive statistics			Goodness-of-fit indicators			
	Mean	Min^a^	Max	MAE	RMSE	ICC	Difference < 0.03 (%)^b^
Observed AQoL-4D utilities	0.702	− 0.040	1.000				
MM_1	0.735	− 0.040	0.908	0.1326	0.1871	0.663	**18.84**
MM_2	0.735	− 0.040	0.931	**0.1325**	0.1872	0.663	18.78
Lasso 3_CV	0.703	− 0.040	0.855	0.1381	**0.1819**	0.646	12.89
Lasso 3_ADP	0.703	− **0.040**	**1.000**	0.1363	0.1827	**0.667**	14.75
SVM_1	0.721	− 0.040	0.903	0.1334	0.1831	**0.667**	17.59

Model 1 was conducted using the AQoL-4D utilities as the dependent variable and the response level of each item (i.e., each WHODAS item has five response levels) as the independent variable. Model 2 was conducted to further control for covariates of age and gender. GLM was performed using the log link and the gamma family. The bolded statistic indicates the best performance results for each goodness-of-fit criterion

*AQoL-4D* Assessment of Quality of Life-4 dimensions, *MM* Robust MM estimator, *GLM* Generalised linear model, *Beta* Beta regression, *SVM* support vector machines, *MAE* mean absolute error, *RMSE* root mean square error, *ICC* intraclass correlation coefficient

^a^Where applicable we truncated the values to be within the interval of the AQoL-4D theoretical boundaries, that is within [− 0.04;1]

^b^The percentage of predicted values that have a difference smaller than 0.03 from the observed AQoL-4D utilities

The Lasso regression in Model 3 using the adaptive method and pairwise interactions of WHODAS responses performed better in predicting the upper and lower boundary of utility (− 0.04 to 1). Like other models, the predicted utilities smaller than − 0.04 were truncated to the AQoL-4D theoretical boundary − 0.04 before the goodness-of-fit calculation. This model and the SVM method in model 1 achieved the highest ICC (ICC = 0.667). The Lasso regression using the cross-validation method in Model 3 performed the best in achieving the lowest RMSE (RMSE = 0.1819). No machine learning techniques we used outperformed the best traditional econometric method with this sample.

Given that the MAE is our primary goodness-of-fit criteria, it is recommended that the MM estimator of Model 2 should be adopted for economic evaluations and other studies looking to predict average utilities for a group of participants.

### Performance in people with different levels of disability restrictions

We examined how well the models performed in predicting the utility for people with different levels of disability restrictions using results from Sect. "Mapping results". The goodness-of-fit assessment (EMS1 Table S5a–e) showed that all models performed worse in predicting the utility for the group with profound or severe core restrictions (*n* = 294, observed mean utility = 0.423) compared to other severity levels, with MAE ranging from 0.1207 to 0.2583. Predicted values over-estimated utilities for this group by 0.069 to 0.165. Models performed better for the groups with mild restriction (*n* = 184) and no specific restrictions (*n* = 1955), with the lowest MAE in these groups.

Table 3 shows that although MM in Model 2 was found to perform the best overall, it did not outperform other models for the other subgroups except for those with no specific restriction (MAE = 0.1207). For the subgroup of profound and severe restrictions, GLM in model 2 with the log function and gamma family outperformed other models and methods (MAE = 0.1581, algorithm presented in ESM 1 Table S6). For the subgroups of moderate restrictions and school/ employment restrictions, the Beta method in model 2 outperformed the other methods (MAE = 0.1459 and 0.1435, respectively, algorithm presented in ESM 1 Table S7). For the subgroup with mild restrictions, the SVM technique in model 1 had the lowest MAE (MAE = 0.1262, STATA codes presented in ESM 1 File 1).

**Table 3:** Goodness-of-fit results for the optimal model in different subgroups

Model specification	Descriptive statistics			Goodness-of-fit indicators			
	Mean	Min^a^	Max	MAE	RMSE	ICC	Difference < 0.03 (%)^b^
Profound/severe restrictions							
Observed value	0.423	− 0.040	1.000				
GLM_2_GAM	0.498	0.112	0.893	0.1619	0.1844	0.702	12.93
Moderate restrictions							
Observed value	0.568	− 0.040	1.000				
Beta_2	0.594	0.020	0.882	0.1487	0.1843	0.660	15.32
Mild restrictions							
Observed value	0.706	0.026	1.000				
SVM_1	0.703	0.068	0.900	0.1262	0.1557	0.699	14.13
School/ employment restriction							
Observed value	0.643	− 0.039	1.000				
Beta_2	0.662	0.073	0.885	0.1450	0.1843	0.604	15.43
No restriction							
Observed value	0.790	− 0.040	1.000				
MM_2	0.809	− 0.040	0.931	0.1207	0.1872	0.418	21.13

Model 1 was conducted using the AQoL-4D utilities as the dependent variable and the response level of each item (i.e., each WHODAS item has five response levels) as the independent variable. Model 2 was conducted to further control for covariates of age and gender. GLM was performed using the log link and the gamma family. The bolded statistic indicates the best performance results for each goodness-of-fit criterion

*AQoL-4D* Assessment of Quality of Life-4 dimensions, *MM* Robust MM estimator, *GLM* Generalised linear model, *Beta* Beta regression, *SVM* support vector machines, *MAE* mean absolute error, *RMSE* root mean square error, *ICC* intraclass correlation coefficient

^a^Where applicable we truncated the values to be within the interval of the AQoL-4D theoretical boundaries, that is within [− 0.04;1]

^b^The percentage of predicted values that have a difference smaller than 0.03 from the observed AQoL-4D utilities

**Fig. 2:**
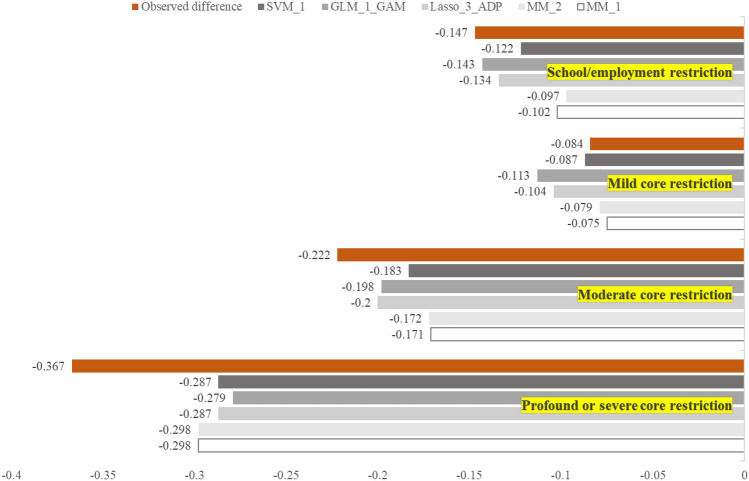
Prediction errors in the average utility differences between different subgroups versus the no restriction group

Figure 2 displays the between-group margins of error. The smallest between-group error was observed in the group with mild core restrictions (*n* = 184, observed between-group mean difference in utilities = − 0.084) and the group with school/employment restrictions (*n* = 473, observed between-group mean difference = − 0.147). The MM estimator had the smallest between-group error only in the group with profound restrictions (error = 0.069). For the subgroups of moderate core restrictions and school/employment restrictions, errors were the smallest when using Lasso regression and GLM, respectively. SVM generated the smallest average errors for the mild restriction group. It is also worth noting that the between-group errors are consistently positive across all subgroups for the MM method. However, they go both positive and negative for some other methods and techniques. More details on the prediction errors of the difference can be found in ESM1 Table S8.

### Mapping algorithms

The final mapping algorithms were then developed using the full sample to increase the precision. The goodness-of-fit results of five selected models using the full study sample are presented in Table S9 in EMS1. The results show that the MM estimator using Model 2 remained the best in the traditional regression methods in achieving the lowest MAE. The error distributions of the predicted values using different methods and models are presented in Fig. S2 in ESM 1.

Table 4 shows the coefficients and standard errors for Model 2 using the MM estimator. The coefficients for Model 1 using MM estimator were also presented for users who do not collect data on age and sex. Based on the reported coefficient in Model 2 using the MM estimator, the predicted utility can be calculated as:

**Table 4:** Optimal mapping equations from WHODAS 2.0–12 to AQoL-4D utilities

Variable	MM2		Variable	MM1	
	Coefficient	SE		Coefficient	SE
wd1_23	− 0.0304064***	0.0073	wd1_23	− 0.0331061***	0.0073
wd1_4	− 0.0582660**	0.0188	wd1_4	− 0.0625948**	0.0190
wd1_5	− 0.1007060***	0.0210	wd1_5	− 0.1053976***	0.0212
wd2_2	− 0.0502851***	0.0097	wd2_2	− 0.0496167***	0.0098
wd2_3	− 0.0765789***	0.0123	wd2_3	− 0.0746950***	0.0126
wd2_4	− 0.1402712***	0.0303	wd2_4	− 0.1387765***	0.0298
wd2_5	− 0.1924466***	0.0365	wd2_5	− 0.1891549***	0.0354
wd3_23	− 0.0220795*	0.0100	wd3_23	− 0.0224716*	0.0101
wd3_45	− 0.0343398	0.0289	wd3_45	− 0.0366910	0.0298
wd4_2	− 0.0211766	0.0114	wd4_2	− 0.0231313*	0.0114
wd4_3	− 0.0519951**	0.0197	wd4_3	− 0.0523269**	0.0199
wd4_4	− 0.1219799**	0.0377	wd4_4	− 0.1253267**	0.0375
wd4_5	− 0.1534310***	0.0337	wd4_5	− 0.1519718***	0.0343
wd5_2	− 0.0520421***	0.0068	wd5_2	− 0.0507621***	0.0067
wd5_3	− 0.0982621***	0.0124	wd5_3	− 0.0992840***	0.0125
wd5_4	− 0.1157922***	0.0204	wd5_4	− 0.1137945***	0.0205
wd5_5	− 0.1668916***	0.0372	wd5_5	− 0.1630724***	0.0386
wd6_2	− 0.0384057***	0.0104	wd6_2	− 0.0347695**	0.0104
wd6_345	− 0.0447633*	0.0180	wd6_3	− 0.0393273*	0.0194
wd7_23	− 0.0093647	0.0080	wd6_45	− 0.0465140	0.0298
wd7_4	− 0.0261744	0.0181	wd7_2	− 0.0037050	0.0092
wd7_5	− 0.0408746**	0.0155	wd7_3	− 0.0294091*	0.0129
wd8_2345	− 0.0391969*	0.0181	wd7_4	− 0.0331811	0.0183
wd9_2	− 0.0454382**	0.0158	wd7_5	− 0.0494096**	0.0155
wd9_345	− 0.0977321**	0.0372	wd8_2345	− 0.0366717*	0.0181
wd11_2	− 0.0529437**	0.0158	wd9_2	− 0.0494831**	0.0157
wd11_3	− 0.0904619**	0.0278	wd9_345	− 0.1023459**	0.0379
wd11_45	− 0.2493463***	0.0431	wd11_2	− 0.0509656**	0.0157
wd12_2	− 0.0404229***	0.0093	wd11_3	− 0.0874626**	0.0281
wd12_3	− 0.0664753***	0.0170	wd11_45	− 0.2424803***	0.0427
wd12_45	− 0.0855047**	0.0289	wd12_2	− 0.0390462***	0.0093
AGE	− 0.0005323***	0.0001	wd12_3	− 0.0623748***	0.0169
SEX	0.0092747	0.0053	wd12_45	− 0.0837476***	0.0294
_cons	0.9270703***	0.0110	_cons	0.9043935***	0.0034

*** *p* < 0.001 **p < 0.01 *p < 0.05; The model was estimated using the MM robust regression controlling for age and sex. The variable column on the left indicates the item number in WHODAS and the level of the item that the coefficient is corresponding to. The number on the left of the underscore denotes the item number, and on the right denotes the level. For example, wd2_2 means level 2 in item 2. Some levels were combined; therefore, they share the same coefficient. For example, levels 3, 4 and 5 of item 6 (wd6_345) share the same coefficient of − 0.0448

AQoL-4D: Assessment of Quality of Life-4 dimensions; WHODAS 2.0–12: World health organisation disability assessment scheme version 2, 12 items; SEX: male = 1

0.9270703-0.0304064*wd1_**_23**_-0.058266*wd1_**_4**_-0.100706*wd1_**_5**_-0.0502851*wd2_**_2**_-0.0765789*wd2_**_3**_-0.1402712*wd2_**_4**_-0.1924466*wd2_**_5**_-0.0220795*wd3_**_23**_-0.0343398*wd3_**_45**_-0.0211766*wd4_**_2**_-0.0519951*wd4_**_3**_-0.1219799*wd4_**_4**_-0.153431*wd4_**_5**_-0.0520421*wd5_**_2**_-0.0982621*wd5_**_3**_-0.1157922*wd5_**_4**_-0.1668916*wd5_**_5**_-0.0384057*wd6_**_2**_-0.0447633*wd6_**_345**_-0.0093647*wd7_**_23**_-0.0261744*wd7_**_4**_-0.0408746*wd7_**_5**_-0.0391969*wd8_**_2345**_-0.0454382*wd9_**_2**_-0.0977321*wd9_**_345**_-0.0529437*wd11_**_2**_-0.0904619*wd11_**_3**_-0.2493463*wd11_**_45**_-0.0404229*wd12_**_2**_-0.0664753*wd12_**_3**_-0.0855047*wd12_**_45**_-0.0005323*Age + 0.0092747*Sex

Each item in WHODAS is denoted by the number after "wd," while the subscripts denote the levels of the item. For instance, "wd2_2" refers to level 2 in item 2. Level 1 (i.e., None) is the reference category. If a male respondent aged 50 selects level 1 for 11 items, except that he experiences moderate difficulty in taking care of household responsibilities (that is he selects level 3 for item 2). Therefore, his utility will be 0.9270703–0.00765789–0.0005323*50 + 0.0092747 = 0.90207211. To establish a monotonic relationship between the items and utilities, we had to combine 13 levels into 10 different levels within several items resulting in 13 of the original level-item variables recombined into 10 variables.^2^ The coefficients for these combined levels for the same item are the same. For example, level 2 and level 3 in Item 1 share the same coefficient of − 0.0583. Item 10 was excluded because it produced a positive coefficient even when all the levels were combined, and its coefficient for the combined item was statistically insignificant.

## Discussion

This study investigated various methods and models to map the WHODAS 2.0-12 items to the AQoL-4D utilities, a generic and well-validated instrument whose utilities could be used in various settings. It allows the estimation of utilities when responses of only WHODAS are collected, which facilitates future economic evaluation of disability interventions.

The mapping study is conducted using people with disability in Australia aged 16–85. Therefore, the observed utility of this sample is lower than the Australian norm for AQoL (0.81, 95%CI 0.81–0.82), which is derived from the same survey we use. Notably, there is only one value set for AQoL-4D, which was developed in an Australian population. Therefore, at the moment, regardless of where the respondents are based globally, the identical Australian-specific value set is used. We understand that generalising the Australian preferences to other countries may not be ideal, but using the Australian value set does not suggest that the instrument could only be valid in the country of development. Previous research showed that between-country variations in value sets may stem from the types of respondent (e.g., proxy vs. self-reported), the methods (e.g., DCE vs. standard gamble), and the composition of the sample selected to do the value tasks [43]. Similar to Health Utilities Index, which developed the value set using only the Canadian sample but have been applied to research globally [44], we believe that AQoL-4D could be applied to broader than the Australian population.

The recommended mapping algorithm identified for a general disability population uses the MM estimator in Model 2, controlling for sex and age. The MAE of the cross-validation samples for this method is 0.1325, which is comparable with the ranges of other mapping studies whose MAE falls between 0.011 and 0.19 [19, 20, 33, 35]. Although the MM estimate overpredicted the utilities, our subgroup analysis showed that, unlike other methods, which over-predicted in some groups but under-predicted in others, the MM estimator consistently over-predicted the utilities across all the subgroups with different restriction levels (hence the prediction errors on the differences between different sub-groups could be minimised). Therefore, when using this recommended algorithm to estimate utilities in economic evaluations, the researchers should be aware that the actual utilities are possibly lower than the estimated ones for people with all levels of restrictions.

Economic evaluations focus on comparing a group receiving an intervention with a group that does not. As the MM estimator in Model 2 overpredicts utilities for all subgroups, the overpredicted errors would be offset when incremental utilities are calculated for comparisons between subgroups. Because the MM estimator generated consistently overestimates between-group differences, researchers should also be cautious while using MM in economic evaluations that compare subgroups with different restriction levels as the true differences may be smaller.

The machine learning technique did not outperform the traditional methods, even though the data-driven approaches allow for more flexibility. This is consistent with other mapping studies using machine-learning techniques [21, 22]. Additionally, controlling for age and sex did not enhance the performance of the machine learning technique, likely because the complexity of variable interactions is already taken into account. Some research has indicated that age and sex may not be statistically significant [34]. However, since age is statistically significant at the 5% level and some of our traditional models included the optimal MM model, we still include it in our algorithm. Sex is only significant at the 10% level in some of our models using traditional methods, but we have also included it because they are commonly included in mapping studies [19, 22, 34], can increase precision for our prediction, and we are not sure if the gender difference will affect the responses to WHOAS and AQoL-4D disproportionally in other data. It should also be noted that the sex variable in our data is a binary variable consisting of female and male. Newer surveys are likely to allow individuals to classify themselves in other categories. Future studies should explore whether gender classifications with more identity choices will impact the precision of the results.

We found that different models and methods predicted different subgroups better. We recommend using MM in model 2 to estimate utilities for a population with different disability levels or people with disability but no specific restrictions. GLM with the log function and gamma family, Beta regression method, and SVM in model 1 are recommended for sensitivity analysis if the sample is concentrated on people with profound/severe, moderate or school/employment, and mild restrictions, respectively. Researchers could use the algorithms and STATA codes provided in the ESM to perform additional sensitivity analysis.

Unique items were identified in our concept mapping process for WHODAS and AQoL-4D, and these items are more likely to require combining levels to achieve monotonic correlation. For example, items related to cognition that were only asked in WHODAS required level combining and an item in WHODAS asking about dealing with unknown people was dropped due to positive correlation. This suggests that the relationship question in WHODAS was focused on a different type of relationship compared to AQoL-4D, which emphasises relationships with friends and family. It highlights the importance of considering the algorithm’s applicability when evaluating interventions.

Several limitations to this study should be acknowledged. The model performance of the study was validated using five-fold cross-validation but with data from the same study sample. Since the mapping is a data-driven exercise, the choice of response sample could affect the calibration of the mapping algorithm. Ideally, in the future, validation will also be possible using an external dataset. The second limitation of the study is that the predicted AQoL-4D utilities under-predict the highest utilities. This is a commonly reported limitation in many mapping studies [33, 35]. However, because the algorithm consistently under-predicts the highest utility in each subgroup, the issue may be attenuated when comparing different groups in an economic evaluation study. The last limitation was that information about the disability types (e.g., physical, psychosocial) was not in the data. However, given the large sample size, we saw many variations in both the source and target instruments, and we were able to perform a subgroup analysis based on the restriction level of disability.

The results from this mapping study indicate that it is reasonable to map WHODAS onto the AQoL-4D utilities. The availability of this mapping algorithm will facilitate future economic evaluation for disability interventions when only the WHODAS is used.

### Supplementary Information

Below is the link to the electronic supplementary material.

Supplementary file1 (DOCX 816 KB)

## Footnotes

1 Between group margins of error = (the predicted average utility value of the restriction subgroup—the predicted average utility value of the no restriction subgroup)—(the observed utility value of the restriction subgroup—the observed utility value of the no restriction subgroup).

2 Level 2 = Level 3 in item1, 3, 7; level 4 = level 5 in item 3, 11, 12; level 3 = level4 = level 5 in item 6, 9; level 2 = level 3 = level 4 = level 5 in item 8.
