# Supplementary material for: Mapping the 12-item World Health Organization disability assessment schedule 2.0 (WHODAS 2.0) onto the assessment of quality of life (AQoL)-4D utilities
Source: Qual Life Res. 2023 Oct 31;33(2):411–22. doi: 10.1007/s11136-023-03532-9 (PMC10850031; doi:10.1007/s11136-023-03532-9)
Supplement: Supplementary file 1 — Supplementary file1 (DOCX 816 KB) [file 11136_2023_3532_MOESM1_ESM.docx]

**Electronic Supplementary Materials 1 (ESM 1)**

**Title:** Mapping the 12-item World Health Organization Disability Assessment Schedule 2.0 (WHODAS 2.0) to the Assessment of Quality of Life 4D (AQOL-4D) utilities

**Journal Name:** Quality of Life Research

**Table S1: A comparison between AQoL-4D and WHODAS 2.0-12**

|  | **AQoL-4D** | **WHODAS 2.0-12** | **Dimensions** |  |
| --- | --- | --- | --- | --- |
| **Descriptive System** | | | |  |
| **Dimensions** | 4 | 6 |  |  |
|  |  |  |  |  |
| **Items** | 12 | 12 |  |  |
| **Response Levels** | 4 | 5 |  |  |
| **Score range** | -0.04, 1 (Higher better) | 12, 60 (Lower better) |  |  |
| **Length of recall period** | 7 days | 30 days |  |  |
| **Dimension Type** | | | |  |
|  | AQoL 3 Get around the house and community | WD1 Standing for long periods | Mobility /Life activities |  |
|  |  | WD7 Walking a long distance WD12 Day-to-day work  WD4 Joining community activities |  |  |
|  | AQoL l 1 Look after yourself | WD8 Washing whole body | Self-care |  |
|  |  | WD9 Getting dressed |  |  |
|  | AQoL 2 Household tasks | WD2 Household responsibilities | Household tasks |  |
|  | AQoL 10 Sleep | WD5 Emotionally affected by health problem | Mental Health |  |
|  | AQoL 11 General feeling |  |  |  |
|  | AQoL 4 Relationships | WD10 Deal with people don't know | Relationships | |
|  | AQoL 5 Friends and loneliness | S11 Maintaining relationships |  |  |
|  | AQoL 6 Health and family relationship |  |  |  |
|  | AQoL 7 Vision |  | Senses |  |
|  | AQoL 8 Hearing |  |  |  |
|  | AQoL 9 Communication difficulties |  |  |  |
|  | AQoL 12 Pain |  |  |  |
|  |  | WD3 Learn new task | Cognition |  |
|  |  | WD6 Concentrate for 10 minutes |  |  |
| **Notes:** Colours in the Dimension Type areas indicate the results from the factor analysis. Items with loadings greater than 0.3 were included in a factor. Green colour indicates factor 1, and orange colour factor 2.   AQoL-4D: Assessment of Quality of Life-4 dimensions; WHODAS 2.0-12: World Health Organisation Disability Assessment Scheme version 2, 12 items; AQoL: AQoL-4D items; WD: WHODAS items. | | | |  |

**Table S2: Correlation coefficients between the items and summary score of WHODAS 2.0-12 and AQoL-4D**

|  | **WD1** | **WD2** | **WD3** | **WD4** | **WD5** | **WD6** | **WD7** | **WD8** | **WD9** | **WD10** | **WD11** | **WD12** | **WD_sum** |
| --- | --- | --- | --- | --- | --- | --- | --- | --- | --- | --- | --- | --- | --- |
| **AQoL1** | 0.2812 | 0.3641 | 0.2191 | 0.2706 | 0.2004 | 0.1607 | 0.3069 | **0.4119** | **0.4511** | 0.1264 | 0.1364 | 0.3175 | **0.4198** |
| **AQoL2** | **0.4453** | **0.5947** | 0.2711 | 0.3413 | 0.3113 | 0.2160 | **0.4653** | 0.3691 | 0.3873 | 0.1440 | 0.1664 | **0.4304** | **0.5648** |
| **AQoL3** | **0.4034** | 0.4418 | 0.2658 | **0.4197** | 0.2411 | 0.2019 | **0.4722** | **0.4135** | **0.4327** | 0.1709 | 0.1597 | **0.4283** | **0.5444** |
| **AQoL4** | 0.1183 | 0.2106 | 0.1622 | 0.2683 | 0.2998 | 0.2603 | 0.1156 | 0.1247 | 0.1266 | 0.2307 | 0.3072 | 0.2408 | 0.3148 |
| **AQoL5** | 0.1899 | 0.2993 | 0.2319 | 0.3977 | **0.4582** | 0.3337 | 0.1561 | 0.1567 | 0.1577 | 0.3444 | **0.4621** | 0.3305 | **0.4520** |
| **AQoL6** | 0.2622 | 0.3929 | 0.2428 | 0.3909 | **0.4431** | 0.3673 | 0.2317 | 0.2443 | 0.2388 | 0.2980 | 0.3982 | **0.4051** | **0.5088** |
| **AQoL7** | 0.1774 | 0.1859 | 0.1958 | 0.1964 | 0.2054 | 0.1662 | 0.1686 | 0.1351 | 0.1442 | 0.1558 | 0.1360 | 0.1832 | 0.2712 |
| **AQoL8** | 0.1098 | 0.0474 | 0.0778 | 0.0866 | 0.0467 | 0.0680 | 0.0807 | 0.0638 | 0.0752 | 0.0366 | 0.0492 | 0.0556 | 0.1069 |
| **AQoL9** | 0.1240 | 0.1524 | 0.2391 | 0.2623 | 0.2258 | 0.2865 | 0.0772 | 0.1324 | 0.1462 | 0.3090 | 0.3141 | 0.2152 | 0.3067 |
| **AQoL10** | 0.2414 | 0.2545 | 0.1501 | 0.2170 | 0.3385 | 0.2301 | 0.1922 | 0.1373 | 0.1727 | 0.1767 | 0.1640 | 0.2530 | 0.3400 |
| **AQoL11** | 0.1586 | 0.3107 | 0.2502 | 0.3510 | **0.5286** | 0.3682 | 0.1165 | 0.1512 | 0.1607 | 0.3261 | 0.3480 | 0.3503 | **0.4395** |
| **AQoL12** | **0.4475** | 0.3899 | 0.1468 | 0.2409 | 0.3597 | 0.2000 | 0.3974 | 0.2604 | 0.2758 | 0.1268 | 0.1504 | 0.3125 | **0.4621** |
| **AQoL4D** | **-0.4467** | **-0.5315** | -0.3297 | **-0.4871** | **-0.5348** | -0.3886 | **-0.4314** | -0.3421 | -0.3611 | -0.3306 | -0.3853 | **-0.4964** | **-0.6758** |

**Notes:** AQoL-4D: Assessment of quality of life-4 dimensions; WHODAS 2.0-12: World health organisation disability assessment scheme version 2, 12 items; AQoL: AQoL-4D items; WD: WHODAS items; WD_sum: The summary score of WHODAS 2.0-12; AQol4D: The utility score of AQol-4D. Correlation coefficient≥0.4 in bold. All the coefficients in this table are significant at the 5% level.

| **Table S3:**  **Exploratory factor analysis comparing AQoL-4D and WHODAS 2.0 items** | | | | |
| --- | --- | --- | --- | --- |
|  |  | **Factor** | |  |
|  |  | 1 | 2 |  |
| WD7 | Walking long distance | 0.74 |  |  |
| WD1 | Standing long periods | 0.69 |  |  |
| WD9 | Getting dressed | 0.67 |  |  |
| WD8 | Washing whole body | 0.64 |  |  |
| AQoL3 | Independent living: Getting around | 0.62 |  |  |
| AQoL2 | Independent living: Household tasks | 0.61 |  |  |
| WD2 | Household responsibilities | 0.60 |  |  |
| AQoL1 | Independent living: Look after yourself | 0.52 |  |  |
| AQoL12 | Mental health: Pain | 0.49 |  |  |
| WD12 | Day-to-day work | 0.38 |  |  |
| AQoL5 | Relationship: lonely |  | 0.68 |  |
| WD11 | Maintaining friendship |  | 0.67 |  |
| AQoL11 | Mental health: worrying |  | 0.66 |  |
| WD10 | Dealing with people don't know |  | 0.60 |  |
| AQoL4 | Relationship: close relationship |  | 0.57 |  |
| WD5 | Emotions |  | 0.54 |  |
| WD6 | Concentration |  | 0.53 |  |
| AQoL6 | Relationship: family role |  | 0.50 |  |
| WD4 | Joining community activities |  | 0.50 |  |
| AQoL9 | Senses: communication |  | 0.42 |  |
| WD3 | Learning new tasks |  | 0.39 |  |
| WD12 | Day-to-day work |  | 0.37 |  |
| AQoL10 | Mental health: sleeping |  | 0.32 |  |

**Notes**: AQoL-4D: Assessment of quality of life-4 dimensions; WHODAS 2.0-12: World health organisation disability assessment scheme version 2, 12 items; AQoL: AQoL-4D items; WD: WHODAS items.

**Table S4: Goodness-of-fit results on the validation samples for all methods and models (n=3,376)**

| **Model specification** | Descriptive statistics | | |  | Goodness-of-fit indicators | | | |
| --- | --- | --- | --- | --- | --- | --- | --- | --- |
|  | Mean | Min^a^ | Max |  | MAE | RMSE | ICC | Difference <0.03 (%)^b^ |
| **Observed value** | 0.702 | -0.040 | 1.000 |  |  |  |  |  |
|  |  |  |  |  |  |  |  |  |
| **OLS_1** | 0.702 | -0.040 | 0.872 |  | 0.137 | 0.1830 | 0.657 | 14.60 |
| **OLS_2** | 0.702 | -0.040 | 0.894 |  | 0.137 | 0.1839 | 0.652 | 13.86 |
|  |  |  |  |  |  |  |  |  |
| **MM_1** | 0.735 | -0.040 | 0.908 |  | 0.133 | 0.1871 | 0.663 | 18.84 |
| **MM_2** | 0.735 | -0.040 | 0.931 |  | **0.132** | 0.1872 | 0.663 | 18.78 |
|  |  |  |  |  |  |  |  |  |
| **GLM_1_GAU** | 0.703 | 0.069 | 1.000 |  | 0.137 | 0.1831 | 0.656 | 14.28 |
| **GLM_1_GAM** | 0.703 | 0.099 | 0.888 |  | 0.137 | 0.1844 | 0.658 | 14.51 |
| **GLM_2_GAU** | 0.702 | 0.111 | 0.910 |  | 0.138 | 0.1842 | 0.649 | 14.04 |
| **GLM_2_GAM** | 0.702 | 0.119 | 0.888 |  | 0.136 | **0.1818** | 0.667 | 14.13 |
|  |  |  |  |  |  |  |  |  |
| **Beta_1** | NOT CONVERGING | | |  |  |  |  |  |
| **Beta_2** | 0.707 | 0.017 | 0.883 |  | 0.134 | 0.1825 | 0.666 | 14.57 |
|  |  |  |  |  |  |  |  |  |
| **Lasso_1_CV** | 0.702 | -0.040 | 0.930 |  | 0.137 | 0.1823 | 0.655 | 13.33 |
| **Lasso_1_ADP** | 0.702 | -0.040 | 0.955 |  | 0.137 | 0.1826 | 0.658 | 13.24 |
| **Lasso_1_Plugin** | 0.702 | 0.016 | 0.809 |  | 0.149 | 0.1901 | **0.564** | **10.55** |
| **Lasso_2_CV** | 0.702 | -0.040 | 0.958 |  | 0.137 | 0.1822 | 0.658 | 13.45 |
| **Lasso_2_ADP** | 0.702 | -0.040 | 0.981 |  | 0.137 | 0.1825 | 0.659 | 14.01 |
| **Lasso_2_Plugin** | 0.702 | 0.017 | 0.809 |  | 0.149 | 0.1902 | **0.564** | 10.60 |
| **Lasso_3_CV** | 0.703 | -0.040 | 0.855 |  | 0.138 | 0.1819 | 0.646 | 12.89 |
| **Lasso_3_ADP** | 0.703 | -0.040 | 1.000 |  | 0.136 | 0.1827 | 0.667 | 14.75 |
| **Lasso_3_Plugin** | 0.702 | -0.040 | 0.822 |  | 0.146 | 0.1876 | 0.587 | 11.49 |
|  |  |  |  |  |  |  |  |  |
| **Boost_1** | 0.703 | -0.040 | 0.878 |  | 0.137 | 0.1824 | 0.660 | 14.57 |
| **Boost_2** | 0.703 | -0.040 | 0.952 |  | 0.137 | 0.1820 | 0.659 | 13.86 |
|  |  |  |  |  |  |  |  |  |
| **SVM_1** | 0.721 | -0.040 | 0.903 |  | 0.133 | 0.1831 | 0.667 | 17.59 |
| **SVM_2** | 0.718 | -0.040 | 0.973 |  | 0.138 | 0.1872 | 0.639 | 15.46 |

**Notes:** Model 1 (“_1) was conducted using the utilities as the dependent variable, and the response level as the independent variables. Model 2 (“_2) was conducted also controlling for covariates of age and gender. GLM was performed using the log link and the gamma family. Lasso was performed using the cross validation (CV), the adaptive (ADP) and the plugin techniques. Model 3 (“_3) in Lasso means the independent variables also include the pairwise interaction of them. Bolded statistic indicates best performance results for each goodness-of-fit criterion.

OLS: Ordinary least square; MM: Robust MM estimator; GLM: Generalised linear model; Beta: Beta regression; Lasso: Least Absolute Shrinkage and Selection Operator; Boost: Boosted regression; SVM: Support vector machines; MAE: Mean absolute error; RMSE: Root mean square error; ICC: Intraclass correlation coefficient.

a: Where applicable we truncated the values to be within the interval of the AQoL-4D theoretical boundaries, that is within [-0.04;1]

b: The percentage of predicted values that have difference smaller than 0.03 from the observed AQoL-4D utilities.

**Table S5a: Predicted utilities for people with profound or severe restrictions**

| **Subgroup: Profound or severe core restriction (n=294)** | | | | | | | | | | | | |  |
| --- | --- | --- | --- | --- | --- | --- | --- | --- | --- | --- | --- | --- | --- |
|  | Descriptive statistics | | | |  | | Goodness-of fit | | | | | |  |
|  | y | min | max | | |  | MAE | RMSE | ICC | | difference  <0.03 (%) | |  |
| **Observed value** | 0.423 | -0.040 | 1.000 | | |  |  |  |  | |  | |  |
| **OLS_1** | 0.501 | -0.040 | 0.870 | | |  | 0.1645 | 0.1830 | 0.691 | | 13.61 | |  |
| **OLS_2** | 0.503 | -0.019 | 0.883 | | |  | 0.1639 | 0.1839 | 0.695 | | 10.88 | |  |
|  |  |  |  | | |  |  |  |  | |  | |  |
| **MM_1** | 0.512 | -0.040 | 0.908 | | |  | 0.1658 | 0.1871 | 0.712 | | 10.88 | |  |
| **MM_2** | 0.512 | -0.040 | 0.924 | | |  | 0.1659 | 0.1872 | 0.711 | | 11.56 | |  |
|  |  |  |  | | |  |  |  |  | |  | |  |
| **GLM_1_GAU** | 0.507 | 0.082 | 0.892 | | |  | 0.1680 | 0.1831 | 0.685 | | 10.20 | |  |
| **GLM_1_GAM** | 0.497 | 0.112 | 0.888 | | |  | 0.1620 | 0.1844 | 0.703 | | 11.90 | |  |
| **GLM_2_GAU** | 0.508 | 0.130 | 0.894 | | |  | 0.1662 | 0.1842 | 0.686 | | 11.56 | |  |
| **GLM_2_GAM** | 0.498 | 0.112 | 0.893 | | |  | 0.1619 | 0.1844 | 0.702 | | 12.93 | |  |
|  |  |  | |  | | |  |  |  |  | |  |  |
| **Beta_1** | NOT CONVERGING | | | | | | | | | | | | |
| **Beta_2** | 0.498 | 0.031 | 0.875 | | |  | 0.1626 | 0.1843 | 0.713 | | 11.56 | |  |
|  |  |  |  | | |  |  |  |  | |  | |  |
| **Lasso_1_CV** | 0.504 | -0.040 | 0.872 | | |  | 0.1643 | 0.2077 | 0.698 | | 10.54 | |  |
| **Lasso_1_ADP** | 0.501 | -0.040 | 0.877 | | |  | 0.1638 | 0.2074 | 0.699 | | 9.86 | |  |
| **Lasso_1_Plugin** | 0.551 | 0.103 | 0.809 | | |  | 0.1940 | 0.2394 | 0.565 | | 7.48 | |  |
| **Lasso_2_CV** | 0.502 | -0.040 | 0.884 | | |  | 0.1636 | 0.2075 | 0.699 | | 11.22 | |  |
| **Lasso_2_ADP** | 0.500 | -0.040 | 0.892 | | |  | 0.1638 | 0.2075 | 0.699 | | 11.56 | |  |
| **Lasso 2_Plugin** | 0.551 | 0.103 | 0.809 | | |  | 0.1941 | 0.2395 | 0.564 | | 7.48 | |  |
| **Lasso_3_CV** | 0.510 | -0.040 | 0.855 | | |  | 0.1650 | 0.2093 | 0.688 | | 13.27 | |  |
| **Lasso_3_ADP** | 0.488 | -0.040 | 0.875 | | |  | 0.1588 | 0.2083 | 0.713 | | 14.63 | |  |
| **Lasso_3_Plugin** | 0.543 | 0.043 | 0.822 | | |  | 0.1863 | 0.2314 | 0.594 | | 9.18 | |  |
|  |  |  |  | | |  |  |  |  | |  | |  |
| **Boost_1** | 0.499 | -0.040 | 0.878 | | |  | 0.1661 | 0.2082 | 0.692 | | 11.90 | |  |
| **Boost_2** | 0.503 | -0.040 | 0.907 | | |  | 0.1662 | 0.2077 | 0.690 | | 10.88 | |  |
|  |  |  |  | | |  |  |  |  | |  | |  |
| **SVM_1** | 0.507 | -0.040 | 0.900 | | |  | 0.1625 | 0.2063 | 0.710 | | 12.59 | |  |
| **SVM_2** | 0.521 | -0.040 | 0.912 | | |  | 0.1780 | 0.2238 | 0.646 | | 9.52 | |  |

**Notes:** Model 1 (“_1) was conducted using the utilities as the dependent variable, and the response level as the independent variables. Model 2 (“_2) was conducted also controlling for covariates of age and gender. GLM was performed using the log link and the gamma family. Lasso was performed using the cross validation, the adaptive and the plugin techniques. Model 3 (“_3) in Lasso means the independent variables also include the pairwise interaction of them. Bolded statistic indicates best performance results for each goodness-of-fit criterion.

OLS: Ordinary least square; MM: Robust MM estimator; GLM: Generalised linear model; Beta: Beta regression; Lasso: Least Absolute Shrinkage and Selection Operator; Boost: Boosted regression; SVM: Support vector machines; MAE: Mean absolute error; RMSE: Root mean square error; ICC: Intraclass correlation coefficient.

a: Where applicable we truncated the values to be within the interval of the AQoL-4D theoretical boundaries, that is within [-0.04;1]

b: The percentage of predicted values that have difference smaller than 0.03 from the observed AQoL-4D utilities

**Table S5b: Predicted utilities for people with moderate restrictions**

| **Subgroup: Moderate core restriction (n=470)** | | | | | | | | |
| --- | --- | --- | --- | --- | --- | --- | --- | --- |
|  | Descriptive statistics | | |  |  | Goodness-of-fit indicators | | |
|  | y | min | max |  | MAE | RMSE | ICC | difference <0.03 (%) |
| **Observed value** | 0.568 | -0.040 | 1.000 |  |  |  |  |  |
| **OLS_1** | 0.590 | -0.040 | 0.870 |  | 0.1490 | 0.1830 | 0.661 | 12.77 |
| **OLS_2** | 0.593 | -0.040 | 0.883 |  | 0.1499 | 0.1839 | 0.650 | 12.13 |
|  |  |  |  |  |  |  |  |  |
| **MM_1** | 0.618 | -0.040 | 0.908 |  | 0.1490 | 0.1871 | 0.663 | 17.66 |
| **MM_2** | 0.618 | -0.040 | 0.923 |  | 0.1495 | 0.1872 | 0.660 | 17.23 |
|  |  |  |  |  |  |  |  |  |
| **GLM_1_GAU** | 0.595 | 0.069 | 0.892 |  | 0.1512 | 0.1831 | 0.645 | 11.06 |
| **GLM_1_GAM** | 0.592 | 0.099 | 0.888 |  | 0.1527 | 0.1844 | 0.646 | 10.85 |
| **GLM_2_GAU** | 0.597 | 0.126 | 0.894 |  | 0.1511 | 0.1842 | 0.635 | 11.70 |
| **GLM_2_GAM** | 0.592 | 0.098 | 0.893 |  | 0.1525 | 0.1844 | 0.647 | 10.00 |
|  |  |  |  |  |  |  |  |  |
| **Beta_1** | NOT CONVERGING | | | | | | | |
| **Beta_2** | 0.594 | 0.020 | 0.882 |  | 0.1487 | 0.1843 | 0.660 | 15.32 |
| **Lasso_1_CV** | 0.595 | -0.040 | 0.872 |  | 0.1501 | 0.1981 | 0.655 | 10.43 |
| **Lasso_1_ADP** | 0.593 | -0.040 | 0.877 |  | 0.1505 | 0.1987 | 0.655 | 10.43 |
| **Lasso_1_Plugin** | 0.621 | 0.016 | 0.809 |  | 0.1596 | 0.2078 | 0.574 | 10.21 |
| **Lasso_2_CV** | 0.593 | -0.040 | 0.874 |  | 0.1496 | 0.1975 | 0.657 | 11.28 |
| **Lasso_2_ADP** | 0.592 | -0.040 | 0.878 |  | 0.1499 | 0.1980 | 0.658 | 11.49 |
| **Lasso 2_Plugin** | 0.622 | 0.017 | 0.809 |  | 0.1597 | 0.2078 | 0.573 | 10.43 |
| **Lasso_3_CV** | 0.601 | -0.013 | 0.855 |  | 0.1501 | 0.1976 | 0.648 | 11.70 |
| **Lasso_3_ADP** | 0.590 | -0.040 | 0.881 |  | 0.1508 | 0.1993 | 0.663 | 12.13 |
| **Lasso_3_Plugin** | 0.617 | -0.040 | 0.822 |  | 0.1569 | 0.2040 | 0.595 | 11.91 |
|  |  |  |  |  |  |  |  |  |
| **Boost_1** | 0.594 | -0.008 | 0.874 |  | 0.1514 | 0.1976 | 0.655 | 12.13 |
| **Boost_2** | 0.594 | -0.002 | 0.924 |  | 0.1510 | 0.1982 | 0.648 | 12.55 |
|  |  |  |  |  |  |  |  |  |
| **SVM_1** | 0.607 | -0.040 | 0.900 |  | 0.2006 | 0.2006 | 0.658 | 13.83 |
| **SVM_2** | 0.608 | -0.040 | 0.967 |  | 0.2103 | 0.2103 | 0.614 | 13.19 |

**Notes:** Model 1 (“_1) was conducted using the utilities as the dependent variable, and the response level as the independent variables. Model 2 (“_2) was conducted also controlling for covariates of age and gender. GLM was performed using the log link and the gamma family. Lasso was performed using the cross validation, the adaptive and the plugin techniques. Model 3 (“_3) in Lasso means the independent variables also include the pairwise interaction of them. Bolded statistic indicates best performance results for each goodness-of-fit criterion.

OLS: Ordinary least square; MM: Robust MM estimator; GLM: Generalised linear model; Beta: Beta regression; Lasso: Least Absolute Shrinkage and Selection Operator; Boost: Boosted regression; SVM: Support vector machines; MAE: Mean absolute error; RMSE: Root mean square error; ICC: Intraclass correlation coefficient.

a: Where applicable we truncated the values to be within the interval of the AQoL-4D theoretical boundaries, that is within [-0.04;1]

b: The percentage of predicted values that have difference smaller than 0.03 from the observed AQoL-4D utilities.

**Table S5c: Predicted utilities for people with mild restrictions**

| **Subgroup: Mild core restriction (n=184)** | | | | | | | | |
| --- | --- | --- | --- | --- | --- | --- | --- | --- |
|  | Descriptive statistics | | |  | Goodness-of-fit indicators | | | |
|  | y | min | max |  | MAE | RMSE | ICC | difference <0.03 (%) |
| **Observed value** | 0.706 | 0.026 | 1.000 |  |  |  |  |  |
| **OLS_1** | 0.682 | 0.090 | 0.872 |  | 0.1316 | 0.1830 | 0.674 | 10.87 |
| **OLS_2** | 0.681 | 0.104 | 0.880 |  | 0.1325 | 0.1839 | 0.660 | 10.87 |
|  |  |  |  |  |  |  |  |  |
| **MM_1** | 0.715 | -0.040 | 0.908 |  | 0.1298 | 0.1871 | 0.664 | 14.13 |
| **MM_2** | 0.710 | -0.040 | 0.920 |  | 0.1307 | 0.1872 | 0.659 | 14.13 |
|  |  |  |  |  |  |  |  |  |
| **GLM_1_GAU** | 0.679 | 0.154 | 0.978 |  | 0.1317 | 0.1831 | 0.683 | 14.13 |
| **GLM_1_GAM** | 0.677 | 0.177 | 0.888 |  | 0.1343 | 0.1844 | 0.659 | 13.04 |
| **GLM_2_GAU** | 0.677 | 0.182 | 0.891 |  | 0.1347 | 0.1842 | 0.650 | 10.87 |
| **GLM_2_GAM** | 0.676 | 0.176 | 0.892 |  | 0.1348 | 0.1844 | 0.657 | 11.96 |
|  |  |  |  |  |  |  |  |  |
| **Beta_1** | NOT CONVERGING | | | | | | | |
| **Beta_2** | 0.687 | 0.082 | 0.875 |  | 0.1340 | 0.1843 | 0.649 | 10.87 |
| **Lasso_1_CV** | 0.683 | 0.055 | 0.884 |  | 0.1316 | 0.1595 | 0.676 | 11.96 |
| **Lasso_1_ADP** | 0.683 | 0.057 | 0.877 |  | 0.1312 | 0.1599 | 0.679 | 10.87 |
| **Lasso_1_Plugin** | 0.686 | 0.202 | 0.809 |  | 0.1386 | 0.1641 | 0.593 | 7.61 |
| **Lasso_2_CV** | 0.679 | 0.048 | 0.872 |  | 0.1322 | 0.1600 | 0.676 | 11.96 |
| **Lasso_2_ADP** | 0.679 | 0.048 | 0.877 |  | 0.1322 | 0.1606 | 0.677 | 10.87 |
| **Lasso 2_Plugin** | 0.686 | 0.203 | 0.809 |  | 0.1386 | 0.1642 | 0.593 | 7.61 |
| **Lasso_3_CV** | 0.688 | 0.085 | 0.855 |  | 0.1283 | 0.1548 | 0.680 | 9.78 |
| **Lasso_3_ADP** | 0.686 | -0.026 | 1.000 |  | 0.1280 | 0.1568 | 0.706 | 12.50 |
| **Lasso_3_Plugin** | 0.686 | 0.205 | 0.822 |  | 0.1348 | 0.1603 | 0.623 | 5.98 |
|  |  |  |  |  |  |  |  |  |
| **Boost_1** | 0.685 | 0.073 | 0.874 |  | 0.1312 | 0.1601 | 0.679 | 12.50 |
| **Boost_2** | 0.680 | 0.089 | 0.869 |  | 0.1309 | 0.1569 | 0.687 | 10.87 |
|  |  |  |  |  |  |  |  |  |
| **SVM_1** | 0.703 | 0.068 | 0.900 |  | 0.1262 | 0.1557 | 0.699 | 14.13 |
| **SVM_2** | 0.701 | 0.110 | 0.909 |  | 0.1282 | 0.1580 | 0.674 | 10.87 |

**Notes:** Model 1 (“_1) was conducted using the utilities as the dependent variable, and the response level as the independent variables. Model 2 (“_2) was conducted also controlling for covariates of age and gender. GLM was performed using the log link and the gamma family. Lasso was performed using the cross validation, the adaptive and the plugin techniques. Model 3 (“_3) in Lasso means the independent variables also include the pairwise interaction of them. Bolded statistic indicates best performance results for each goodness-of-fit criterion.

OLS: Ordinary least square; MM: Robust MM estimator; GLM: Generalised linear model; Beta: Beta regression; Lasso: Least Absolute Shrinkage and Selection Operator; Boost: Boosted regression; SVM: Support vector machines; MAE: Mean absolute error; RMSE: Root mean square error; ICC: Intraclass correlation coefficient.

a: Where applicable we truncated the values to be within the interval of the AQoL-4D theoretical boundaries, that is within [-0.04;1]

b: The percentage of predicted values that have difference smaller than 0.03 from the observed AQoL-4D utilities.

**Table S5d: Predicted utilities for people with school/employment restrictions**

| **Subgroup: School/employment restriction (n=473)** | | | | | | | | |
| --- | --- | --- | --- | --- | --- | --- | --- | --- |
|  | Descriptive statistics | | |  |  | Goodness-of-fit indicators | | |
|  | y | min | max |  | MAE | RMSE | ICC | difference <0.03 (%) |
| **Observed value** | 0.643 | -0.039 | 1.000 |  |  |  |  |  |
| **OLS_1** | 0.652 | 0.110 | 0.872 |  | 0.1467 | 0.1830 | 0.594 | 15.64 |
| **OLS_2** | 0.655 | 0.095 | 0.894 |  | 0.1463 | 0.1839 | 0.593 | 15.86 |
|  |  |  |  |  |  |  |  |  |
| **MM_1** | 0.688 | -0.008 | 0.906 |  | 0.1448 | 0.1871 | 0.598 | 16.49 |
| **MM_2** | 0.693 | -0.010 | 0.931 |  | 0.1442 | 0.1872 | 0.601 | 16.91 |
|  |  |  |  |  |  |  |  |  |
| **GLM_1_GAU** | 0.649 | 0.158 | 0.888 |  | 0.1467 | 0.1831 | 0.601 | 14.38 |
| **GLM_1_GAM** | 0.646 | 0.179 | 0.888 |  | 0.1479 | 0.1844 | 0.603 | 13.32 |
| **GLM_2_GAU** | 0.652 | 0.158 | 0.910 |  | 0.1453 | 0.1842 | 0.605 | 13.74 |
| **GLM_2_GAM** | 0.663 | 0.180 | 0.896 |  | 0.1474 | 0.1844 | 0.648 | 13.11 |
|  |  |  |  |  |  |  |  |  |
| **Beta_1** | NOT CONVERGING | | | | | | | |
| **Beta_2** | 0.662 | 0.073 | 0.885 |  | 0.1450 | 0.1843 | 0.604 | 15.43 |
| **Lasso_1_CV** | 0.654 | 0.062 | 0.864 |  | 0.1467 | 0.1923 | 0.596 | 13.74 |
| **Lasso_1_ADP** | 0.652 | 0.048 | 0.867 |  | 0.1468 | 0.1923 | 0.599 | 14.16 |
| **Lasso_1_Plugin** | 0.669 | 0.254 | 0.809 |  | 0.1557 | 0.1999 | 0.494 | 10.99 |
| **Lasso_2_CV** | 0.657 | 0.056 | 0.882 |  | 0.1454 | 0.1919 | 0.601 | 15.22 |
| **Lasso_2_ADP** | 0.656 | 0.042 | 0.888 |  | 0.1452 | 0.1917 | 0.605 | 15.86 |
| **Lasso 2_Plugin** | 0.669 | 0.255 | 0.809 |  | 0.1558 | 0.1999 | 0.494 | 10.99 |
| **Lasso_3_CV** | 0.657 | 0.006 | 0.855 |  | 0.1488 | 0.1949 | 0.568 | 13.74 |
| **Lasso_3_ADP** | 0.655 | -0.031 | 0.892 |  | 0.1482 | 0.1963 | 0.590 | 14.38 |
| **Lasso_3_Plugin** | 0.665 | 0.151 | 0.822 |  | 0.1540 | 0.1992 | 0.510 | 11.63 |
|  |  |  |  |  |  |  |  |  |
| **Boost_1** | 0.649 | 0.013 | 0.874 |  | 0.1499 | 0.1969 | 0.584 | 13.53 |
| **Boost_2** | 0.653 | 0.045 | 0.928 |  | 0.1472 | 0.1940 | 0.596 | 14.16 |
|  |  |  |  |  |  |  |  |  |
| **SVM_1** | 0.668 | -0.001 | 0.900 |  | 0.1460 | 0.1955 | 0.598 | 14.59 |
| **SVM_2** | 0.671 | -0.040 | 0.919 |  | 0.1502 | 0.1998 | 0.567 | 14.38 |

**Notes:** Model 1 (“_1) was conducted using the utilities as the dependent variable, and the response level as the independent variables. Model 2 (“_2) was conducted also controlling for covariates of age and gender. GLM was performed using the log link and the gamma family. Lasso was performed using the cross validation, the adaptive and the plugin techniques. Model 3 (“_3) in Lasso means the independent variables also include the pairwise interaction of them. Bolded statistic indicates best performance results for each goodness-of-fit criterion.

OLS: Ordinary least square; MM: Robust MM estimator; GLM: Generalised linear model; Beta: Beta regression; Lasso: Least Absolute Shrinkage and Selection Operator; Boost: Boosted regression; SVM: Support vector machines; MAE: Mean absolute error; RMSE: Root mean square error; ICC: Intraclass correlation coefficient.

a: Where applicable we truncated the values to be within the interval of the AQoL-4D theoretical boundaries, that is within [-0.04;1]

b: The percentage of predicted values that have difference smaller than 0.03 from the observed AQoL-4D utilities.

**Table S5e: Predicted utilities for people with no specific restrictions**

| **Subgroup: No specific restriction (n=1,955)** | | | | | | | | |
| --- | --- | --- | --- | --- | --- | --- | --- | --- |
|  | Descriptive statistics | | |  |  | Goodness-of-fit indicators | | |
|  | y | min | max |  | MAE | RMSE | ICC | difference <0.03 (%) |
| **Observed value** | 0.790 | -0.040 | 1.000 |  |  |  |  |  |
| **OLS_1** | 0.773 | 0.003 | 0.872 |  | 0.1277 | 0.1830 | 0.417 | 15.29 |
| **OLS_2** | 0.772 | -0.040 | 0.894 |  | 0.1280 | 0.1839 | 0.416 | 14.53 |
|  |  |  |  |  |  |  |  |  |
| **MM_1** | 0.810 | -0.040 | 0.908 |  | 0.1209 | 0.1871 | 0.418 | 21.33 |
| **MM_2** | 0.809 | -0.040 | 0.931 |  | 0.1207 | 0.1872 | 0.418 | 21.13 |
|  |  |  |  |  |  |  |  |  |
| **GLM_1_GAU** | 0.774 | 0.109 | 1.000 |  | 0.1277 | 0.1831 | 0.432 | 15.65 |
| **GLM_1_GAM** | 0.776 | 0.099 | 0.888 |  | 0.1277 | 0.1844 | 0.429 | 16.21 |
| **GLM_2_GAU** | 0.772 | 0.111 | 0.910 |  | 0.1284 | 0.1842 | 0.425 | 15.35 |
| **GLM_2_GAM** | 0.776 | 0.099 | 0.900 |  | 0.1278 | 0.1844 | 0.429 | 16.16 |
|  |  |  |  |  |  |  |  |  |
| **Beta_1** | NOT CONVERGING | | | | | | | |
| **Beta_2** | 0.780 | 0.022 | 0.883 |  | 0.1259 | 0.1825 | 0.418 | 15.24 |
| **Lasso_1_CV** | 0.772 | 0.088 | 0.930 |  | 0.1284 | 0.1738 | 0.414 | 14.37 |
| **Lasso_1_ADP** | 0.773 | 0.071 | 0.955 |  | 0.1281 | 0.1741 | 0.416 | 14.42 |
| **Lasso_1_Plugin** | 0.754 | 0.236 | 0.809 |  | 0.1388 | 0.1770 | 0.323 | 11.25 |
| **Lasso_2_CV** | 0.772 | 0.078 | 0.958 |  | 0.1281 | 0.1738 | 0.417 | 14.02 |
| **Lasso_2_ADP** | 0.772 | 0.073 | 0.981 |  | 0.1280 | 0.1742 | 0.418 | 14.83 |
| **Lasso 2_Plugin** | 0.754 | 0.237 | 0.809 |  | 0.1388 | 0.1770 | 0.322 | 11.30 |
| **Lasso_3_CV** | 0.768 | 0.037 | 0.855 |  | 0.1296 | 0.1726 | 0.406 | 13.20 |
| **Lasso_3_ADP** | 0.775 | -0.040 | 0.892 |  | 0.1273 | 0.1731 | 0.427 | 15.70 |
| **Lasso_3_Plugin** | 0.757 | -0.040 | 0.822 |  | 0.1363 | 0.1754 | 0.351 | 12.23 |
|  |  |  |  |  |  |  |  |  |
| **Boost_1** | 0.774 | 0.116 | 0.874 |  | 0.1274 | 0.1727 | 0.435 | 16.01 |
| **Boost_2** | 0.773 | 0.123 | 0.952 |  | 0.1275 | 0.1729 | 0.433 | 14.83 |
|  |  |  |  |  |  |  |  |  |
| **SVM_1** | 0.794 | 0.064 | 0.903 |  | 0.1224 | 0.1742 | 0.433 | 20.31 |
| **SVM_2** | 0.788 | 0.137 | 0.973 |  | 0.1255 | 0.1744 | 0.419 | 17.60 |

**Notes:** Model 1 (“_1) was conducted using the utilities as the dependent variable, and the response level as the independent variables. Model 2 (“_2) was conducted also controlling for covariates of age and gender. GLM was performed using the log link and the gamma family. Lasso was performed using the cross validation, the adaptive and the plugin techniques. Model 3 (“_3) in Lasso means the independent variables also include the pairwise interaction of them. Bolded statistic indicates best performance results for each goodness-of-fit criterion.

OLS: Ordinary least square; MM: Robust MM estimator; GLM: Generalised linear model; Beta: Beta regression; Lasso: Least Absolute Shrinkage and Selection Operator; Boost: Boosted regression; SVM: Support vector machines; MAE: Mean absolute error; RMSE: Root mean square error; ICC: Intraclass correlation coefficient.

a: Where applicable we truncated the values to be within the interval of the AQoL-4D theoretical boundaries, that is within [-0.04;1]

b: The percentage of predicted values that have difference smaller than 0.03 from the observed AQoL-4D utilities.

**Table S6** **Alternative** **mapping equations from WHODAS 2.0-12 to AQoL-4D utilities**

**using the GLM log function and gamma family**

| **Variable** | **Coefficient** | **Standard errors** |
| --- | --- | --- |
| wd1_23 | -0.0437* | 0.0177 |
| wd1_4 | -0.1440* | 0.0316 |
| wd1_5 | -0.1767*** | 0.0337 |
| wd2_2 | -0.0667** | 0.0202 |
| wd2_3 | -0.1009** | 0.0251 |
| wd2_4 | -0.3099*** | 0.0403 |
| wd2_5 | -0.3046*** | 0.0558 |
| wd3_2345 | -0.0128 | 0.0195 |
| wd4_2 | -0.0229 | 0.0241 |
| wd4_3 | -0.0760* | 0.0309 |
| wd4_45 | -0.3136*** | 0.0338 |
| wd5_2 | -0.0805*** | 0.0176 |
| wd5_3 | -0.1533*** | 0.0220 |
| wd5_45 | -0.2784*** | 0.0285 |
| wd6_2345 | -0.0618** | 0.0186 |
| wd7_234 | -0.0038 | 0.0185 |
| wd7_5 | -0.0953** | 0.0280 |
| wd8_2345 | -0.0419 | 0.0332 |
| wd9_2 | -0.1005** | 0.0318 |
| wd9_345 | -0.1722*** | 0.0459 |
| wd11_2 | -0.0887** | 0.0276 |
| wd11_3 | -0.2540*** | 0.0375 |
| wd11_45 | -0.3241*** | 0.0525 |
| wd12_2 | -0.0097 | 0.0204 |
| wd12_3 | -0.1152*** | 0.0272 |
| wd12_45 | -0.2065*** | 0.0389 |
| SEX | -0.0017 | 0.0138 |
| Age | -0.0003 | 0.0004 |
| Constant | -0.1144*** | 0.0248 |

**Notes:** *** p<0.001 **p<0.01 *p<0.05; The model was estimated using the GLM log function and gamma family controlling for age and sex. The variable column on the left indicates the item number in WHODAS and the level of the item that the coefficient is corresponding to. The number on the left of the underscore denotes the item number, and on the right denotes the level. For example, wd2_2 means level 2 in item 2. Some levels were combined; therefore, they share the same coefficient. For example, levels 3, 4 and 5 of item 6 (wd6_345) shared the same coefficient of -0.0448.

AQoL-4D: Assessment of Quality of Life-4 dimensions; WHODAS 2.0-12: World health organisation disability assessment scheme version 2, 12 items version; Sex: male=1

**Table S7 Alternative** **mapping equations from WHODAS 2.0-12 to AQoL-4D utilities**

**using Betamix regression**

| **Variable** | **Coefficient** | **Standard errors** |
| --- | --- | --- |
| C1_mu |  |  |
| wd1_23 | -0.418035*** | 0.1110459 |
| wd1_4 | -0.7687587*** | 0.1973194 |
| wd1_5 | -0.9186187*** | 0.2121769 |
| wd2_2345 | -0.3282421** | 0.1237068 |
| wd3_2345 | -0.0687914 | 0.1373372 |
| wd4_2345 | -0.2571131 | 0.134279 |
| wd5_2 | -0.7611691*** | 0.1132567 |
| wd5_3 | -0.8856021*** | 0.1455218 |
| wd5_45 | -1.269893*** | 0.1804533 |
| wd6_2 | -0.1196206 | 0.1479187 |
| wd6_345 | -0.4684523** | 0.1805191 |
| wd7_2345 | -0.1246936 | 0.1135457 |
| wd9_2345 | -0.0583005 | 0.1622678 |
| wd11_2 | -0.4000808* | 0.1956405 |
| wd11_345 | -0.5327875** | 0.1983695 |
| wd12_23 | -0.3815128** | 0.1330347 |
| wd12_45 | -0.4153922 | 0.2333662 |
| SEX | 0.0465431 | 0.0834175 |
| AGE | -0.0026794 | 0.0023284 |
| _cons | 2.479235*** | 0.1496342 |
| C1_lnphi |  |  |
| _cons | 0.3066039*** | 0.0576821 |
| C2_mu |  |  |
| wd1_23 | -0.1248493** | 0.0414872 |
| wd1_45 | -0.2739445*** | 0.0626143 |
| wd2_23 | -0.2773537*** | 0.0426668 |
| wd2_45 | -0.6820186*** | 0.0877737 |
| wd3_2345 | -0.0707408 | 0.0457758 |
| wd4_2 | -0.100527 | 0.053466 |
| wd4_3 | -0.2450553*** | 0.0696139 |
| wd4_45 | -0.7122288*** | 0.0915185 |
| wd5_2 | -0.2349069*** | 0.0403324 |
| wd5_3 | -0.4922268*** | 0.0532989 |
| wd5_45 | -0.5146628*** | 0.0724088 |
| wd6_2 | -0.164752** | 0.0498333 |
| wd6_345 | -0.2072742** | 0.0690043 |
| wd7_23 | -0.055496 | 0.044765 |
| wd7_45 | -0.2054586*** | 0.0556346 |
| wd9_2 | -0.2389417*** | 0.0597991 |
| wd9_345 | -0.780954*** | 0.1236597 |
| wd11_2 | -0.2082842** | 0.0658817 |
| wd11_3 | -0.4147737*** | 0.0950625 |
| wd11_45 | -1.221563*** | 0.1417957 |
| wd12_2 | -0.160463*** | 0.0458603 |
| wd12_345 | -0.2588712*** | 0.0635506 |
| SEX | 0.0258434 | 0.0323362 |
| AGE | -0.0032465*** | 0.0009234 |
| _cons | 1.918405*** | 0.0597389 |
| C2_lnphi |  |  |
| _cons | 2.460338*** | 0.0494934 |
| Prob_C1 |  |  |
| _cons | -0.9812592*** | 0.0755638 |
| C1_phi | 1.358803 | 0.0783787 |
| C2_phi | 11.70877 | 0.5795073 |
| pi1 | 0.272642 | 0.0149849 |
| pi2 | 0.727358 | 0.0149849 |

**Notes:** *** p<0.001 **p<0.01 *p<0.05; The model was estimated using the Betamix regression controlling for age and sex. The variable column on the left indicates the item number in WHODAS and the level of the item that the coefficient is corresponding to. The number on the left of the underscore denotes the item number, and on the right denotes the level. For example, wd2_2 means level 2 in item 2. Some levels were combined; therefore, they share the same coefficient. For example, levels 3, 4 and 5 of item 6 (wd6_345) shared the same coefficient of -0.0448.

AQoL-4D: Assessment of Quality of Life-4 dimensions; WHODAS 2.0-12: World health organisation disability assessment scheme version 2, 12 items version; Sex: male=1

**Table S8: Utilities difference between groups different disability severity levels vs. no restrictions**

| **Ref group:  No restriction** | **Profound or severe core restriction^a^ (n=294)** | **Error of the predicted  difference^b^** | **Moderate core restriction^a^ (N=470)** | **Error of the predicted difference^b^** | **Mild core restriction^a^ (n=184)** | **Error of the predicted difference^b^** | **School/ employment restriction^a^ (n=473)** | **Error of the predicted difference^b^** |
| --- | --- | --- | --- | --- | --- | --- | --- | --- |
| **Observed value** | -0.367 |  | -0.222 |  | -0.084 |  | -0.147 |  |
| **OLS_1** | -0.272 | 0.095 | -0.199 | **0.022** | -0.107 | -0.023 | -0.137 | 0.009 |
| **OLS_2** | -0.269 | 0.098 | -0.196 | 0.026 | -0.108 | -0.024 | -0.135 | 0.012 |
|  |  |  |  |  |  |  |  |  |
| **MM_1** | -0.298 | 0.070 | -0.171 | 0.050 | -0.075 | 0.009 | -0.102 | 0.045 |
| **MM_2** | -0.298 | **0.069** | -0.172 | 0.050 | -0.079 | 0.005 | -0.097 | 0.050 |
|  |  |  |  |  |  |  |  |  |
| **GLM_1_GAU** | -0.267 | 0.100 | -0.195 | 0.027 | -0.111 | -0.026 | -0.141 | 0.006 |
| **GLM_1_GAM** | -0.279 | 0.088 | -0.198 | 0.024 | -0.113 | -0.028 | -0.143 | **0.003** |
| **GLM_2_GAU** | -0.264 | 0.103 | -0.193 | 0.029 | -0.113 | -0.029 | -0.138 | 0.009 |
| **GLM_2_GAM** | -0.280 | 0.087 | -0.199 | 0.023 | -0.115 | -0.031 | -0.142 | 0.005 |
|  |  |  |  |  |  |  |  |  |
| **Beta_1** | NOT CONVERGING | | |  |  |  |  |  |
| **Beta_2** | -0.283 | 0.084 | -0.197 | 0.025 | -0.104 | -0.020 | -0.128 | 0.019 |
|  |  |  |  |  | -0.790 | -0.706 | -0.790 | -0.643 |
| **Lasso_1_CV** | -0.268 | 0.099 | -0.195 | 0.027 | -0.106 | -0.022 | -0.136 | 0.011 |
| **Lasso_1_ADP** | -0.271 | 0.096 | -0.197 | 0.025 | -0.106 | -0.022 | -0.137 | 0.009 |
| **Lasso_1_Plugin** | -0.202 | 0.165 | -0.168 | 0.054 | -0.104 | -0.020 | -0.121 | 0.026 |
| **Lasso_2_CV** | -0.270 | 0.098 | -0.196 | 0.026 | -0.110 | -0.026 | -0.133 | 0.014 |
| **Lasso_2_ADP** | -0.272 | 0.095 | -0.198 | 0.024 | -0.111 | -0.027 | -0.134 | 0.013 |
| **Lasso 2_Plugin** | -0.202 | 0.165 | -0.168 | 0.054 | -0.104 | -0.020 | -0.121 | 0.026 |
| **Lasso_3_CV** | -0.258 | 0.109 | -0.189 | 0.033 | -0.102 | -0.017 | -0.133 | 0.014 |
| **Lasso_3_ADP** | -0.287 | 0.080 | -0.200 | **0.022** | -0.104 | -0.020 | -0.134 | 0.012 |
| **Lasso_3_Plugin** | -0.213 | 0.154 | -0.173 | 0.049 | -0.104 | -0.020 | -0.125 | 0.022 |
|  |  |  |  |  |  |  |  |  |
| **Boost_1** | -0.275 | 0.092 | -0.195 | 0.026 | -0.104 | -0.020 | -0.140 | 0.006 |
| **Boost_2** | -0.271 | 0.096 | -0.196 | 0.026 | -0.110 | -0.025 | -0.136 | 0.010 |
|  |  |  |  |  |  |  |  |  |
| **SVM_1** | -0.287 | 0.080 | -0.183 | 0.039 | -0.087 | **-0.003** | -0.122 | 0.025 |
| **SVM_2** | -0.267 | 0.100 | -0.181 | 0.040 | -0.089 | -0.005 | -0.119 | 0.028 |
| **Notes:** Model 1 (“_1) was conducted using the utilities as the dependent variable, and the response level as the independent variables. Model 2 (“_2) was conducted also controlling for covariates of age and gender. GLM was performed using the log link and the gamma family. Lasso was performed using the cross validation, the adaptive and the plugin techniques. Model 3 (“_3) in Lasso means the independent variables also include the pairwise interaction of them. Bolded statistic indicates best performance results for each goodness-of-fit criterion.   OLS: Ordinary least square; MM: Robust MM estimator; GLM: Generalised linear model; Beta: Beta regression; Lasso: Least Absolute Shrinkage and Selection Operator; Boost: Boosted regression; SVM: Support vector machines; MAE: Mean absolute error; RMSE: Root mean square error; ICC: Intraclass correlation coefficient.  a: The utility difference between the disability severity group specified vs. no restriction. Calculated by using the utility value in the disability severity group minus the utility value in the no restriction group in each of the model b: The error of predicted difference comparing to the observed difference of the utilities of different disability severity group vs. no restriction. Calculated by using the value in a minus the observed value corresponding to each disability severity group. | | | | | | | | |

| **Table S9: Goodness-of-fit results from full sample (n=3,376)** | | | | | | | | |
| --- | --- | --- | --- | --- | --- | --- | --- | --- |
| **Model specification** | Descriptive statistics | | |  | Goodness-of-fit indicators | | | |
|  | Mean | Min^a^ | Max |  | MAE | RMSE | ICC | Difference <0.03 (%)^b^ |
| **Observed value** | 0.702 | -0.040 | 1.000 |  |  |  |  |  |
|  |  |  |  |  |  |  |  |  |
| **MM_1** | 0.734 | -0.040 | 0.904 |  | 0.1305 | 0.1852 | 0.670 | 19.28 |
| **MM_2** | 0.734 | -0.040 | 0.928 |  | 0.1304 | 0.1854 | 0.669 | 19.22 |
| **Lasso 3_CV** | 0.702 | -0.020 | 0.903 |  | 0.1322 | 0.1736 | 0.679 | 12.65 |
| **Lasso 3_ADP** | 0.702 | -0.040 | 1.000 |  | 0.1282 | 0.1713 | 0.705 | 15.46 |
| **SVM_1** | 0.721 | -0.040 | 0.902 |  | 0.1271 | 0.1749 | 0.697 | 17.74 |
| **Notes:** Model 1 (_1) was conducted using the utilities as the dependent variable, and the response level as the independent variables. Model 2 (_2) was conducted also controlling for covariates of age and gender. Model 3 (_3) was conducted with pair-wise interactions of the WHODAS item levels. Bolded statistic indicates best performance results for each goodness-of-fit criteria among all the models and methods.   AQoL-4D: Assessment of Quality of Life-4 dimensions; MM: Robust MM estimator; GLM: Generalised linear model; Beta: Beta regression; SVM: Support vector machines; MAE: Mean absolute error; RMSE: Root mean square error; ICC: Intraclass correlation coefficient.  a: Where applicable we truncated the values to be within the interval of the AQoL-4D theoretical boundaries, that is within [-0.04;1]  b: The percentage of predicted values that have difference smaller than 0.03 from the observed AQoL-4D utilities | | | | | | | | |
|  |  |  |  |  |  |  |  |  |
|  |  |  |  |  |  |  |  |  |
|  |  |  |  |  |  |  |  |  |
|  |  |  |  |  |  |  |  |  |
|  |  |  |  |  |  |  |  |  |
|  |  |  |  |  |  |  |  |  |

**Fig S1: Sample selection**


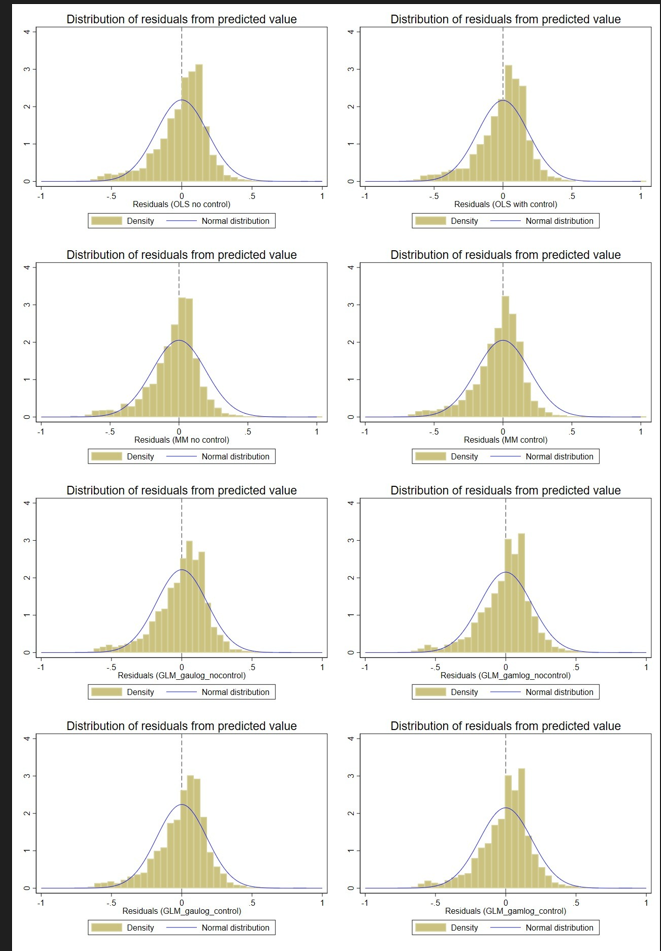


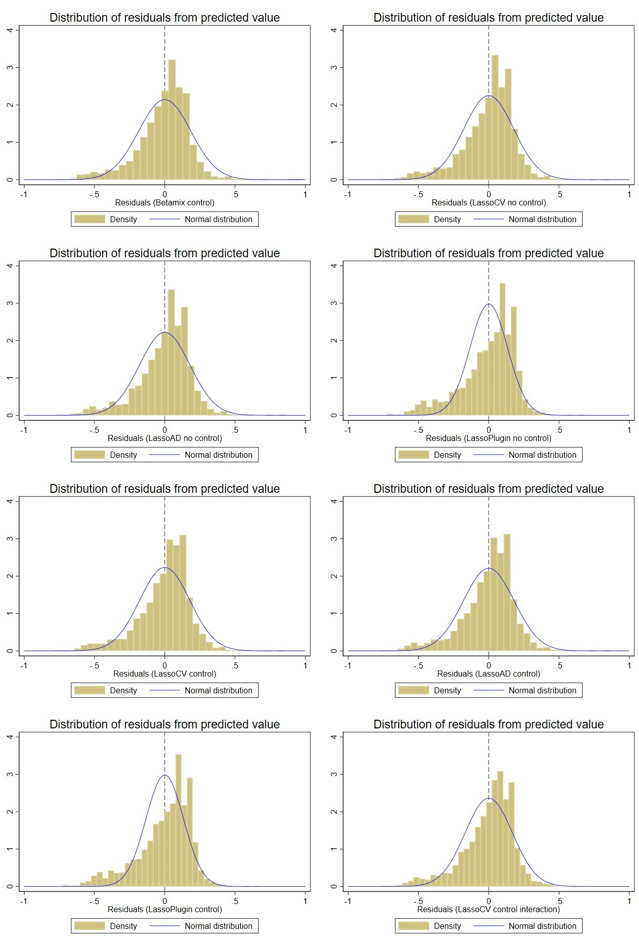


**
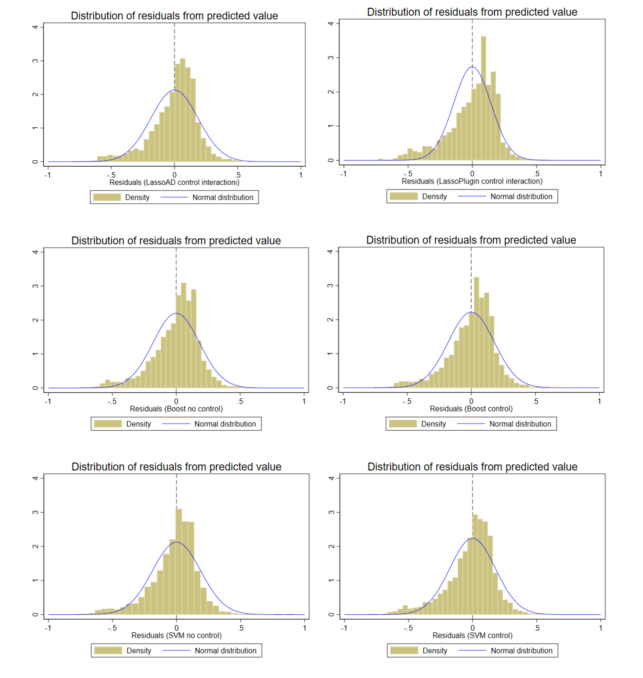
**

**Fig S2 Distribution of prediction errors of different methods and models^[[1]](#footnote-1)^**

**EMS 1 File 1**

**STATA code for Lasso adaptive in Model 3**

global WHODAS_lvld " wd1_2 wd1_3 wd1_4 wd1_5 wd2_2 wd2_3 wd2_4 wd2_5 wd3_2 wd3_3 wd3_4 wd3_5 wd4_2 wd4_3 wd4_4 wd4_5 wd5_2 wd5_3 wd5_4 wd5_5 wd6_2 wd6_3 wd6_4 wd6_5 wd7_2 wd7_3 wd7_4 wd7_5 wd8_2 wd8_3 wd8_4 wd8_5 wd9_2 wd9_3 wd9_4 wd9_5 wd10_2 wd10_3 wd10_4 wd10_5 wd11_2 wd11_3 wd11_4 wd11_5 wd12_2 wd12_3 wd12_4 wd12_5"

global interact "i.($WHODAS_lvld)##i.($WHODAS_lvld)"

set seed 6568

lasso linear uAQoL4D $interact SEX AGECURF, nolog rseed(8568769) selection(adaptive)

lassocoef, display(coef, postselection)

**STATA code for SVM in Model 1**

global WHODAS_lvld " wd1_2 wd1_3 wd1_4 wd1_5 wd2_2 wd2_3 wd2_4 wd2_5 wd3_2 wd3_3 wd3_4 wd3_5 wd4_2 wd4_3 wd4_4 wd4_5 wd5_2 wd5_3 wd5_4 wd5_5 wd6_2 wd6_3 wd6_4 wd6_5 wd7_2 wd7_3 wd7_4 wd7_5 wd8_2 wd8_3 wd8_4 wd8_5 wd9_2 wd9_3 wd9_4 wd9_5 wd10_2 wd10_3 wd10_4 wd10_5 wd11_2 wd11_3 wd11_4 wd11_5 wd12_2 wd12_3 wd12_4 wd12_5"

set seed 85498

svmachines uAQoL4D $WHODAS_lvld, type(svr) sv(svi)

1. “No control” refers to Model 1 in which sex and age are not controlled for. “Control” refers to Model 2 in which sex and age are controlled for. [↑](#footnote-ref-1)
